# A Novel Hybrid Monte Carlo Algorithm for Sampling Path Space

**DOI:** 10.3390/e23050499

**Published:** 2021-04-22

**Authors:** Francis J. Pinski

**Affiliations:** Department of Physics, University of Cincinnati, Cincinnati, OH 45221, USA; frank.pinski@uc.edu; Tel.: +1-513-432-8717

**Keywords:** Brownian dynamics, stochastic processes, sampling path space, transition paths

## Abstract

To sample from complex, high-dimensional distributions, one may choose algorithms based on the Hybrid Monte Carlo (HMC) method. HMC-based algorithms generate nonlocal moves alleviating diffusive behavior. Here, I build on an already defined HMC framework, hybrid Monte Carlo on Hilbert spaces (Beskos, et al. *Stoch. Proc. Applic.* 2011), that provides finite-dimensional approximations of measures π, which have density with respect to a Gaussian measure on an infinite-dimensional Hilbert (path) space. In all HMC algorithms, one has some freedom to choose the mass operator. The novel feature of the algorithm described in this article lies in the choice of this operator. This new choice defines a Markov Chain Monte Carlo (MCMC) method that is well defined on the Hilbert space itself. As before, the algorithm described herein uses an enlarged phase space Π having the target π as a marginal, together with a Hamiltonian flow that preserves Π. In the previous work, the authors explored a method where the phase space π was augmented with Brownian bridges. With this new choice, π is augmented by Ornstein–Uhlenbeck (OU) bridges. The covariance of Brownian bridges grows with its length, which has negative effects on the acceptance rate in the MCMC method. This contrasts with the covariance of OU bridges, which is independent of the path length. The ingredients of the new algorithm include the definition of the mass operator, the equations for the Hamiltonian flow, the (approximate) numerical integration of the evolution equations, and finally, the Metropolis–Hastings acceptance rule. Taken together, these constitute a robust method for sampling the target distribution in an almost dimension-free manner. The behavior of this novel algorithm is demonstrated by computer experiments for a particle moving in two dimensions, between two free-energy basins separated by an entropic barrier.

## 1. Introduction

Often, it is important to understand how molecules change conformations. For example, the folding of proteins is of high interest [1]. One approach is to simulate such transitions and generate a thermodynamic-relevant ensemble of paths that start in one free-energy basin and end in another. The molecular motion here is assumed to be driven by the random thermal motions of the surroundings. This is modeled by Brownian dynamics with the thermal noise supplied by a heat reservoir operating at the fixed temperature ϵ.

The evolution of the particle position *x* is described by the Stochastic Differential Equation (SDE):

(1) $$dx_{t}\;=\;F\left(x\right)\;dt\;+\;\sqrt{2\;\epsilon \;}\;dW_{t}$$

where $dW_{t}$ is the standard Wiener process. Here, the force (drift) is assumed to be the (negative) gradient of a potential energy function U(x), namely F(x)=−∂U/∂x. The physical potential U(x) must be bounded from below to be physical.

Integrating the SDE forward over time, one assembles a trajectory: the starting position may be known, but not the ending point. As shown by Onsager and Machlup [2], the path probability can be expressed in terms of the path positions. The (negative) logarithm of this probability has become known as the Onsager–Machlup (OM) functional. As shown in Appendix A, the form of the functional depends on how the SDE (Equation (1)) is discretized. To understand molecular transitions, it would be useful to extract a thermodynamic distribution of paths that start in one free energy basin and end in another. For such constrained paths, an effective Hamiltonian can be formed and expressed as:

(2) $$H_{eff}=\frac{1}{2}\langle x\;\;|\;L\;|\;\;x\rangle +\Phi \left(x\right),$$

with the details given in Appendix A. Using this effective Hamiltonian, standard techniques can be used to sample the probability distribution at an effective temperature $\epsilon_{eff}=2\;\epsilon /\Delta t$, with ϵ being the physical temperature and Δt being the time discretization step used in approximating the SDE.

Performing such path sampling is challenging for several reasons. Firstly, as the time step used to solve the SDE becomes small, that is as Δt→0, contributions from the high-frequency modes lead to a divergence when evaluated using the effective Hamiltonian (Equation (2)). Secondly, at each point along the path, one must have a full description of the molecule. Thirdly, barriers separating different pathways may be insurmountable. Thus, it is extremely important to have efficient algorithms to probe the thermodynamic-relevant distribution of paths. The work by Beskos et al. [3] addressed the first of these issues using a Hybrid Monte Carlo (HMC) [4] method that augments the path space with Brownian bridges. One of the advantages is that HMC generates nonlocal moves, alleviating the diffusive behavior that plagues other methods [5]. However, in that method, the velocities form a Brownian bridge, and thus, they scale with the (time) path length. This growth negatively affects the accuracy of the algorithm. As shown in Section 2, by creating velocities that form an Ornstein–Uhlenbeck (OU) [6] bridge, one can eliminate this dependence on the path length, while continuing to treat the high-frequency modes accurately. In Section 2, the algorithm for the deterministic integration in the Molecular Dynamics (MD) step is described, as well as the associated error in the effective energy. In Section 3, an example in two dimensions is described, as well as how the algorithmic parameters are chosen. In the novel scheme described here, the time step for the deterministic, molecular dynamics integration can be increased by at least an order of magnitude over the previous method [3], leading to a concomitant decrease in computational effort. In other words, the novel method presented here is at least 10 times faster. This is followed in Section 4 by the description of the results for sampling paths that contain a transition from one basin to another. In Section 5, the continuous-time limit of the OM functional is sampled. As found before [7,8], this form produces unphysical results. The paper ends with a short discussion of how this new algorithm can be used in calculations employing the path integral molecular dynamic method and, finally, with some concluding remarks.

## 2. Time Evolution of the Hamiltonian

In sampling the probability distribution, the effective Hamiltonian, Equation (2), is augmented by including Gaussian distributed variables that can be identified as the momentum, with the aim of using a Hybrid (or Hamiltonian) Monte Carlo (HMC) method. The method is summarized as follows: first, pick the augmented variables from their known distribution; use them to (approximately) evolve the Hamiltonian flow; and then, accept or reject the evolved path using the Metropolis–Hastings criterion. The augmented Hamiltonian is given by:

(3) $$H=\frac{1}{2}\langle p\;\;|\;{\left(L+A_{ou}^{2}\;𝟙\right)}^{-1}\;|\;\;p\rangle +\frac{1}{2}\langle q\;\;|\;L\;|\;\;q\rangle +\Phi \left(q\right).$$

The braket notation, introduced in Appendix A, is used to simplify the look of the equations. Define $x=q+l_{t}$ as the physical path with $l_{t}$ being a term linear in time that allows *x* to have the starting point $x\left(0\right)=x_{-}$ and the ending point $x\left(T\right)=x_{+}$. Note that $\Phi \left(x\right)=\Phi \left(q+l_{t}\right)$ and is denoted as Φ(q). The path $l_{t}$ is given by the linear (in *t*) relationship: $l_{t}=x_{-}+t\;\left(x_{+}-x_{-}\right)/T$. The path *p* is the momentum conjugate to the path *q*. The distribution of the momentum *p* is known, but not transparent. However, the distribution of the velocities is easier to understand, as it corresponds to an OU process, with $A_{ou}$ being the OU parameter.

To understand how the momenta *p* are distributed, one begins with the OU process:

(4) $$dv_{t}\;=\;-A_{ou}\;v_{t}\;dt+\sqrt{2\;\epsilon \;}\;dW_{t}.$$

The OM functional for this process is:

(5) $$I_{om}\;\propto \;\frac{1}{2}\langle v\;|\;(L+A_{ou}^{2}\;𝟙\rangle |v>+\mathrm{unimportant}\mathrm{constant}.$$

The mass operator can be defined as $M=(L+A_{ou}^{2}\;𝟙)$ and then the momentum as |p>=M|v>, giving the effective Hamiltonian for the momentum variables as:

(6) $$\begin{array}{c} H_{ou}=\frac{1}{2}\langle p\;\;|\;M^{-1}\;|\;\;p\rangle \end{array}$$

Thus, the velocities will correspond to an OU process with both endpoints being zero, which is an OU bridge. See Appendix B for the numerical details for constructing realizations of OU bridges.

Now return to the original Hamiltonian. Equation (3) with some modifications is:

(7) $$\begin{array}{c} H_{\alpha }=\frac{1}{2}\langle p\;\;|\;M^{-1}\;|\;\;p\rangle +\frac{1}{2}\langle q\;\;|\;M\;|\;\;q\rangle +\alpha \left(\Phi \left(q\right)-\frac{1}{2}\langle q\;\;|\;A_{ou}^{2}𝟙\;|\;\;q\rangle \right). \end{array}$$

Note that with α=1, this Hamiltonian (Equation (7)) has the term $\frac{1}{2}\langle q\;\;|\;A_{ou}^{2}𝟙\;|\;\;q\rangle$ added and then subtracted. This algebraic manipulation importantly simplifies the numerical procedure, as will be shown below. The role (and value) of the parameter α will be addressed later.

It is convenient to work with the velocities *v* given by $|v>=M^{-1}|p>$, which comprise a bridge being zero at both endpoints. When $A_{ou}=0$, the velocities form a Brownian bridge, with covariance (with t≥s) being $\;E\;\left(v_{s}\;\times \;v_{t}\right)=s\left(1-t/T\right)$ where *T* is the length of the path. This gives $\;E\;v_{T/2}^{\;2}=T/4$, which grows with the (time) length of the path. In contrast, the covariance of an OU bridge is given by:

(8) $$E\;v_{s}\;v_{t}=\frac{2\;\epsilon }{A_{ou}}\frac{\sinh\;(A_{ou}s)\;\sinh\;(A_{ou}\left(T-t\right))}{\sinh\;(A_{ou}T)}\;\mathrm{with}t\geq s,$$

with ϵ being the temperature. At the midpoint of the OU bridge, $\;E\;v_{T/2}^{2}\approx \epsilon /A_{ou}$ when $A_{ou}T\gg 2$, which is independent of the path length *T*. In Appendix B, the numerical procedure for constructing an OU bridge is described.

Defining ϕ in terms of Φ as ϕ(q)=∂Φ/∂q, the equations of motion are given by:

(9) $$\left(\begin{array}{c} \frac{d}{dt}|q>\\ M\frac{d}{dt}|p> \end{array}\right)=\underset{A}{\underset{︸}{\left(\begin{array}{c} \;|v>\\ -M\;|q> \end{array}\right)}}+\underset{B}{\underset{︸}{\alpha \;\left(\begin{array}{c} \;0\\ A_{ou}^{2}\;\left|q>-\right|\phi \left(q\right)> \end{array}\right)}}\;$$

with $\left|\phi \left(q\right)>=\{\phi \left(q_{0}\right),\;\phi \left(q_{1}\right),\;\phi \left(q_{3}\right),\;...\;,\phi \left(q_{N_{t}}\right)\right\}$ and with the *A* and *B* groupings being used in the splitting implemented in the numerical algorithms. This method used is symplectic as it corresponds to a symmetric splitting in a Trotter–Strang [9,10] procedure.

### 2.1. Splitting: ABA

The numerical implementation of the ABA splitting is given in this section. The BAB splitting is relegated to Appendix C and Appendix D. Schematically, the integration can be viewed as pictured in Equation (10). The ABA procedure begins with the initial position and velocity $\left(q_{0},\;v_{0}\right)$, then during the time interval h/2, these evolve into $\left(q_{H},\;v_{H}\right)$ using Equations (11) and (12). Next is the so-called full step, where the velocity evolves over a time *h* changing to $w_{H}$ using Equation (13). The final transformation takes $\left(q_{H},\;w_{H}\right)$ and turns them into $\left(q_{1},\;v_{1}\right)$ during a time interval h/2 using Equations (14) and (15).

(10) $$\left(\begin{array}{c} q_{0}\\ v_{0} \end{array}\right)\overset{A(h/2)}{\underset{\;\mathrm{Half}\;\mathrm{step}}{\Rightarrow }}\left(\begin{array}{c} q_{H}\\ v_{H} \end{array}\right)\overset{B(h)\;}{\underset{\;\mathrm{Full}\;\mathrm{step}}{\Rightarrow }}\left(\begin{array}{c} q_{H}\\ w_{H} \end{array}\right)\overset{A(h/2)}{\underset{\;\mathrm{Half}\;\mathrm{step}}{\Rightarrow }}\left(\begin{array}{c} q_{1}\\ v_{1} \end{array}\right)$$

Half step, A: $\left\{q_{0},\;v_{0}\right\}\Rightarrow \left\{q_{H},\;v_{H}\right\}$ with $\theta =\frac{h}{2}$:

(11) $$\frac{d}{dt}\;|q>=\;\;|v>\;\Rightarrow \;|q_{H}>=\;\;\cos\;(\theta )\;\left|q_{0}>+\sin\;(\theta )\;\right|v_{0}>$$

(12) $$\frac{d}{dt}\left|v>=-|q>\;\Rightarrow \;\right|v_{H}>=-\sin\;(\theta )\;\left|q_{0}>+\cos\;(\theta )\;\right|v_{0}>$$

Full step, B: $\left\{q_{H},\;v_{H}\right\}\Rightarrow \left\{q_{H},\;w_{H}\right\}$ with $M=(L+A_{ou}^{2}𝟙)$:

(13) $$M\;\frac{d}{dt}\left|v>=-\alpha \;\left(|\phi \left(q\right)>-A_{ou}^{2}|q>\right)\;\Rightarrow M\right|\Delta \bar{v}>=-\alpha \;h\;\left(|\phi \left(q_{H}\right)>-A_{ou}^{2}|q_{H}>\right)$$

with $|\Delta \bar{v}>=\left|w_{H}>-\;\right|v_{H}>$.

Half step, A: $\left\{q_{H},\;w_{H}\right\}\Rightarrow \left\{q_{1},\;v_{1}\right\}$ with $\theta =\frac{h}{2}$:

(14) $$\frac{d}{dt}\left|q>=\;\;|v>\;\Rightarrow \;\right|q_{1}>=\;\;\cos\;(\theta )\;\left|q_{H}>+\sin\;(\theta )\;\right|w_{H}>$$

(15) $$\frac{d}{dt}\left|v>=-|q>\;\Rightarrow \;\right|v_{1}>=-\sin\;(\theta )\;\left|q_{H}>+\cos\;(\theta )\;\right|w_{H}>$$

Note that the integrations in the half steps are exact, while the full step integration is approximate. As the numerical algorithm is based on a symmetric splitting of the time evolution operator, it is symplectic and represents the flow of a shadow Hamiltonian [11,12]. Thus, for a small integration time step *h*, the energy error will be bounded. In the following section, the formula for this error is examined.

### 2.2. “Energy” Error

The deterministic MD integration is preformed over a total time $N_{MD}\;h$. For each time increment, the integration corresponds to the ABA method described above. The total energy error for the MD integration can be determined from the sum of the errors of each ABA step. Thus, it is sufficient to find the expression for the energy error in a single ABA step.

The error of the “energy” in the ABA step is due to the middle (full) Step B, since the first and third steps reflect exact integrations. The error is given by:

(16) $$\begin{array}{c} \begin{array}{cc} \Delta E^{\left(01\right)}= & \;\Phi \left(q_{1}\right)-\Phi \left(q_{0}\right)-\frac{1}{2}\langle q_{1}\;\left|\;A_{ou}^{2}\;\right|\;q_{1}\rangle +\frac{1}{2}\langle q_{1}\;\left|\;A_{ou}^{2}\;\right|\;q_{0}\rangle \\ + & \;\frac{1}{2}\langle v_{1}\;\left|\;M\;\right|\;v1\rangle -\frac{1}{2}\langle v_{0}\;\left|\;M\;\right|\;v_{0}\rangle +\frac{1}{2}\langle q_{1}\;\left|\;M\;\right|\;q1\rangle -\frac{1}{2}\langle q_{0}\;\left|\;M\;\right|\;q_{0}\rangle \end{array} \end{array}$$

which simplifies to:

(17) $$\begin{array}{c} \Delta E^{\left(01\right)}=\Phi \left(q_{1}\right)-\Phi \left(q_{0}\right)-\frac{1}{2}\langle q_{1}+q_{0}\;\left|\;A_{ou}^{2}\;\right|\;q_{1}-q_{0}\rangle +\frac{1}{2}\langle w_{H}+v_{H}\;\left|\;M\;\right|\;w_{H}-v_{h}\rangle . \end{array}$$

Substituting for $v_{H}$ and $w_{H}$, one transforms the last term using:

(18) $$\langle w_{H}+v_{H}\;\left|\;M\;\right|\;w_{H}-v_{h}\rangle =-\frac{h\;\alpha }{\sin\theta }\langle \;\phi \left(q_{h}\right)-A_{ou}^{2}q_{H}\;|\;q_{1}-q_{0}\;\rangle$$

The value of α can be chosen to be α=sinc(h/2). With this value of α, the energy error is given as:

(19) $$\Delta E^{\left(01\right)}=\Phi \left(q_{1}\right)-\Phi \left(q_{0}\right)\;-\langle \;\phi \left(q_{H}\right)-A_{ou}^{2}\left(q_{h}-{\bar{q}}_{01}\right)\;|\;q_{1}-q_{0}\;\rangle$$

with ${\bar{q}}_{01}=\left(q_{1}+q_{0}\right)/2$. Now, it is clear why α was chosen in this way. The approximate equations of motion reproduce the flow of a shadow Hamiltonian [11,12]. Evidently, the flow produced by numerically solving the equations of motion with α=sinc(h/2) is closer to the actual flow than to the one with unit α.

The full step in the ABA splitting is:

(20) $$M\;|\Delta v>=-\alpha \;h\;\left(\;|\phi \left(q_{H}\right)>-A_{ou}^{2}|q_{H}>\right)$$

The operator $M^{-1}$ is defined with the boundary conditions such that Δv is zero at both the beginning and end of the path. The above is then a matrix equation: with *M* being a symmetric, real tridiagonal matrix, with the only nonzero elements given by:

(21) $$M_{i,\;i}=A_{ou}^{2}+\frac{2}{\Delta t^{2}}\;\;M_{i,\;i+1}=M_{i+1,\;i}=-\frac{1}{\Delta t^{2}}.$$

With the vanishing of Δv at both ends of the path, there are no endpoint corrections to *M*. To solve the matrix equation, the standard Gaussian elimination procedure is used (without pivoting).

## 3. Numerical Experiments

**Figure 1:**
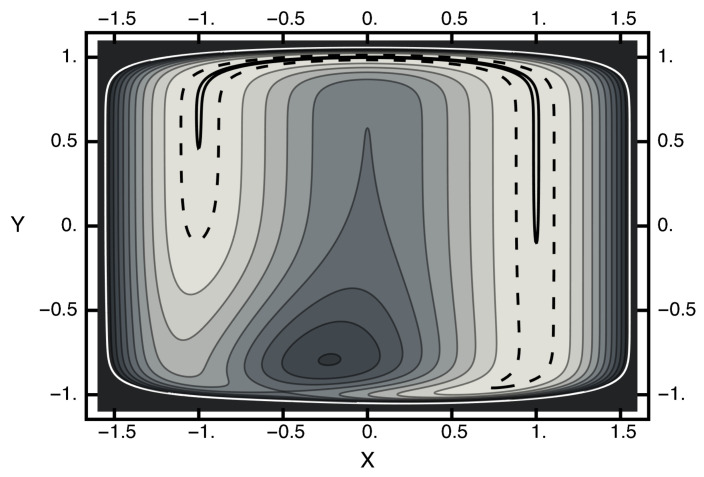
Contour plot of the two-dimensional potential. The horizontal axis corresponds to the *x* value; the vertical axis, the *y* axis. The value of the white contour is two and of the dashed contour is 0.05. The solid black contour enclosed by the dashed contour has a value of 0.001. The potential at the saddle point is approximately unity. The potential at the local maximum is ≈1.6.

To explore the efficacy of the HMC method described above, I chose a two-dimensional example. A particle moving in a potential is given by:

(22) $$\begin{array}{c} U\left(x,\;y\right)\;=\;\exp\;\left(\;-2\;{\left(x+\frac{1}{2}\right)}^{2}-3\;{\left(y+1\right)}^{2}\;\;\right)+{\left(x^{2}+y^{16}-1\right)}^{2} \end{array}$$

where *x* and *y* are the usual Cartesian coordinates. The first term above is a unit Gaussian centered at $x_{g}=-0.5$ and $y_{g}=-1$. The second term is a trough, with a minimum of zero and somewhat squarish in shape. A contour plot of this potential is given in Figure 1. In this figure, note that the white contour corresponds to U(x,y)=2 and the dashed line designates the contour U[x,y)=0.05, which encloses the solid black contour U(x,y)=0.001. A saddle point exists near $x_{s}\approx -0.5$ and $y_{s}\approx -1$ with $U[x_{s},\;y_{s})=1$. A local maximum exists near $x_{m}\approx -0.225$ and $y_{m}\approx -0.794$ with $U(x_{m},\;y_{m})\approx 1.6$. Connecting the free-energy basins is an energy-barrier-free path that becomes severely narrowed as it crosses the x-axis. Thus, one can view this potential as consisting of two basins separated by an entropic barrier if the temperature is not too high.

### 3.1. Equilibrium Distribution

**Figure 2:**
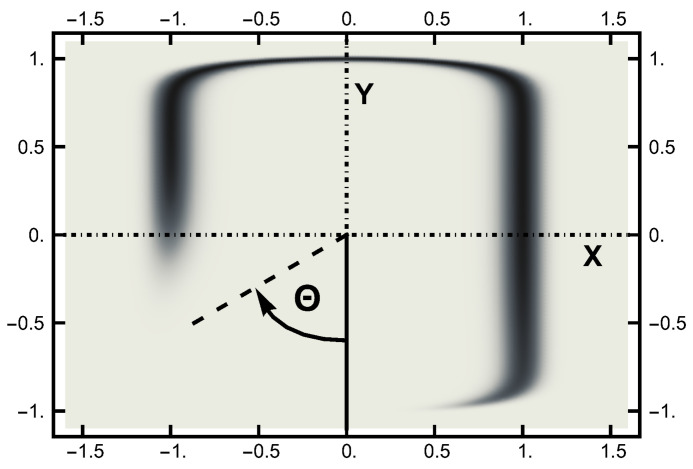
The equilibrium (Boltzmann) distribution for a temperature ϵ=0.05 plotted as a “density” plot. The black areas denote the higher probability areas. The narrow channel connects the left and right probability basins. The lack of an energy barrier is seen by the lack of variation in the shading along the channel. The angle Θ is pictured here.

In this work, the computer experiments were performed with a temperature ϵ=0.05. The temperature is much smaller than the lowest energy barrier, which is unity at the saddle point. The equilibrium (Boltzmann) distribution at a temperature ϵ=0.05 is displayed in the next two figures. In Figure 2, this distribution is shown in terms of a density plot. The narrow channel at the top of the figure connects the smaller basin on the left with the larger basin (on the right). The lack of an energy barrier is reflected in the (equal) values of the distribution along the channel.

**Figure 3:**
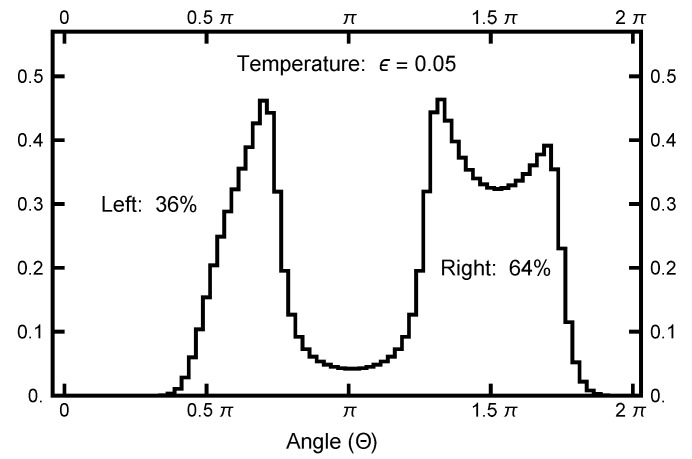
The function $\bar{P}\left(\Theta \right)$ plotted as a function of the angle Θ. See Equation (23) for its definition. The value of δ=π/40 was used. Notice that the single-peak structure on the left differs from the twin peaks on the right. This structure is caused by the geometric factor, which arises when the radius line slices the distribution function.

To quantify the equilibrium distribution, I looked at its values as a function of angle Θ, which is defined in a clockwise sense, measured with respect to the negative *y* axis, as defined in Figure 2. At a temperature ϵ=0.05, the left basin has 36% of the weight, and the remaining 64% is in the right basin. A function $\bar{P}$ is defined as

(23) $$\bar{P}\left(\Theta \right)={\left(Z\;\delta \right)}^{-1}\;\;\;\int_{\Theta -\delta /2}^{\Theta +\delta /2}\;\;\;\;\;d\theta \int_{0}^{\infty }\;\;\;\;\;r\;dr\;\exp\;\left(-\frac{U(-r\;\sin\theta ,\;r\;\cos\theta )}{\epsilon }\right),$$

and is plotted in Figure 3 to emphasize the entropic barrier between the two basins.

### 3.2. Parameter Tuning

Unless stated otherwise, the form of the OM functional used was that corresponding to the midpoint integration of the SDE as written in Equation (A9). To investigate the two-dimensional paths that contain a transition between the two basins, one must pick several parameters; some are physical: the temperature and the path length. For this problem, the temperature was chosen to be ϵ=0.05. This temperature is small compared to the unit energy barrier. Thus, at this temperature, the narrowness of the channel will be the limiting factor that impedes basin hopping. The path length *T* must not be too long nor too short. For a very short value of *T*, the motion will become ballistic and uninteresting. For a very large *T*, the path will be dominated by intra-basin motion, which is not the focus of the study. The remaining parameters are those of the algorithm: the time length of the deterministic integration, the OU bridge parameters, $A_{ou}^{x}$ and $A_{ou}^{y}$, and finally, the value of the algorithmic time step *h*. The last of these will be determined by requiring the Metropolis–Hastings acceptance rate to be sufficiently large.

#### 3.2.1. Path Length

**Figure 4:**
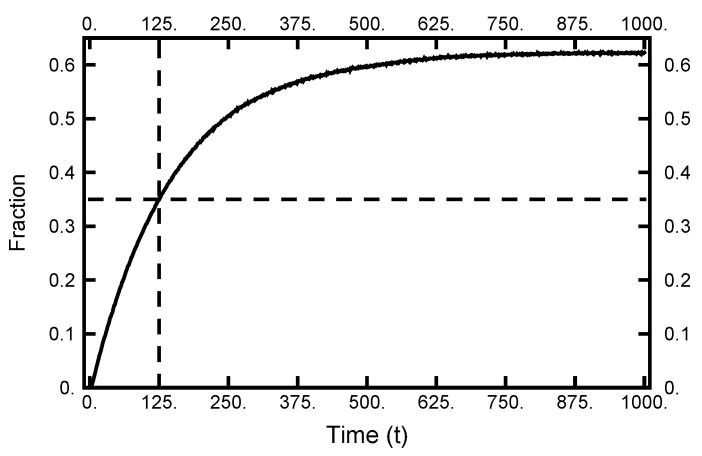
The results of 10,000 forward integrations of Brownian dynamics with temperature ϵ=0.05. All had the same initial conditions (in the left basin). The plot gives the fraction of paths with positive values of *x* as a function of time. As the dotted lines indicate, after T=125, over 35% of the integrations have ending points in the right well. As the integration time exceeds 1000, the fraction approaches the equilibrium distribution.

The next point to address is how quickly the particle moves from the left to right basins. To make an estimate of this transition time, I collected information from 10,000 realizations of Brownian dynamics by performing forward integrations of the two-dimensional SDE. Each integration started at the same place in the left basin. The results for the number of attempts residing in the right basin are tabulated as a function of time and plotted in Figure 4. As one sees, the curve approaches its equilibrium value as the total time reaches 1000. Furthermore, when the elapsed time is 125, over a third of the realizations are in the right basin. Thus, this latter value is a reasonable time (path length) to use for exploring doubly constrained paths that contain a transition. In the following sections, paths are examined that start in the left basin and end in the right basin, while time T=125 has elapsed. The path is then described by 125,001 ordered pairs with $\Delta t=1.0\times 10^{-3}$.

#### 3.2.2. Deterministic Integration Time

**Figure 5:**
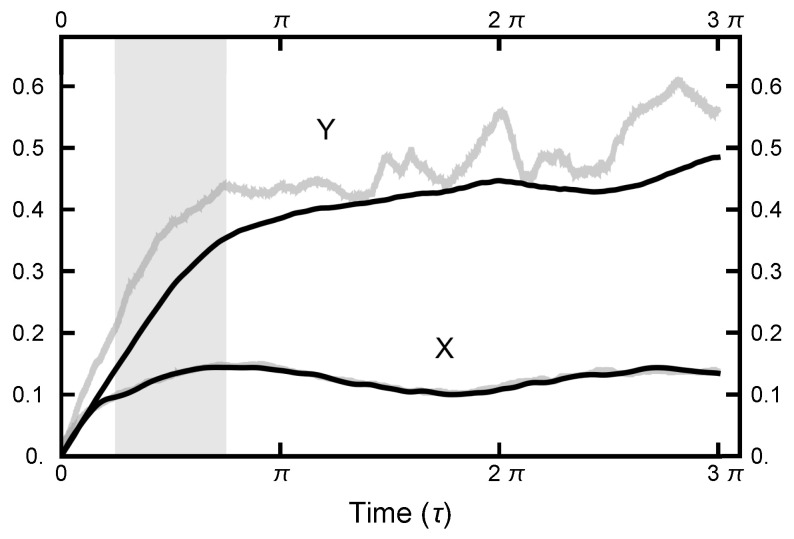
The correlation functions $d^{\left(x\right)}\left(\tau \right)$ and $d^{\left(y\right)}\left(\tau \right)$ for two runs. For the black curves, the parameters were $A_{OU}^{x}=A_{OU}^{y}=1$; for the gray curves, $A_{OU}^{x}=A_{OU}^{y}=0$. The functions $d^{\left(x\right)}\left(\tau \right)$ are almost identical for the two runs: the lower black curve lies on top of the lower gray curve, hiding it.

**Figure 6:**
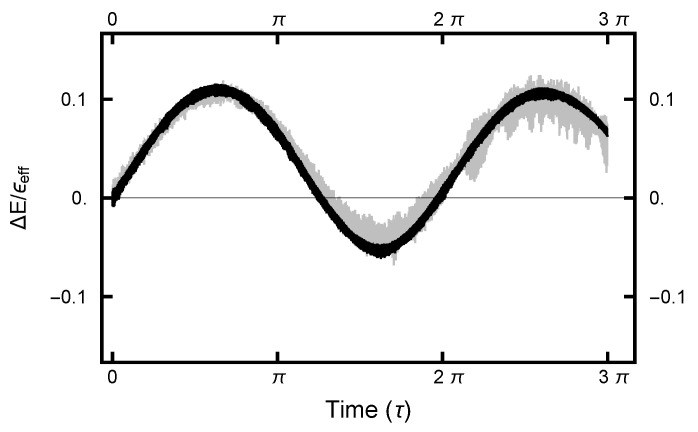
The energy error plotted as a function of MD integration time. The black curves designate the error for the case $A_{ou}^{\left(x\right)}=A_{ou}^{\left(y\right)}=\;1$; the gray curves for $A_{ou}^{\left(x\right)}=A_{ou}^{\left(y\right)}=\;0$. The time step parameter for the former case is $h=6.667\times 10^{-4}$ and for the latter $h=6.667\times 10^{-5}$. In the latter case, the smaller *h* meant that an order of magnitude more computing resources were required to generated the gray curve, as compared to the black one.

One of the advantages of the HMC method is its ability to generate nonlocal moves. To explore the nonlocality of a proposed move, the correlation function $d^{\left(x\right)}\left(n\;h\right)$ is defined by:

(24) $$d^{\left(x\right)}\left(n\;h\right)=\sqrt{\frac{\sum_{i=1}^{N_{t}}{\left(x_{i}\left(n\;h\right)-x_{i}\left(0\right)\right)}^{2}}{N_{t}}}$$

with a similar definition for $d^{\left(y\right)}\left(n\;h\right)$, where *n* is the number of the molecular dynamics steps and $N_{t}$ is the number of points along the path. In Figure 5, $d^{\left(x\right)}\left(\tau \right)$ and $d^{\left(y\right)}\left(\tau \right)$ are plotted as functions of τ for two cases, $A_{ou}^{\left(x\right)}=A_{ou}^{\left(y\right)}=\;0$ and $A_{ou}^{\left(x\right)}=A_{ou}^{\left(y\right)}=\;1$. The time step parameter *h* was adjusted so that the (energy) errors were comparable, as seen in Figure 6. Such energy errors will be examined more closely later in the paper. As seen in Figure 5, both $d^{\left(x\right)}\left(\tau \right)$ and $d^{\left(y\right)}\left(\tau \right)$ level out for τ>2. Thus, the MD integration time should be on the order of π/2 to maximize the nonlocality of the move. To avoid any resonances that might occur if a fixed integration time should be chosen, the length of the integration is chosen to be uniformly distributed in the interval (π/4,3π/4) designated by the gray shaded area in Figure 5.

#### 3.2.3. OU Bridge Parameter $A_{ou}^{\left(x,\;y\right)}$

**Figure 7:**
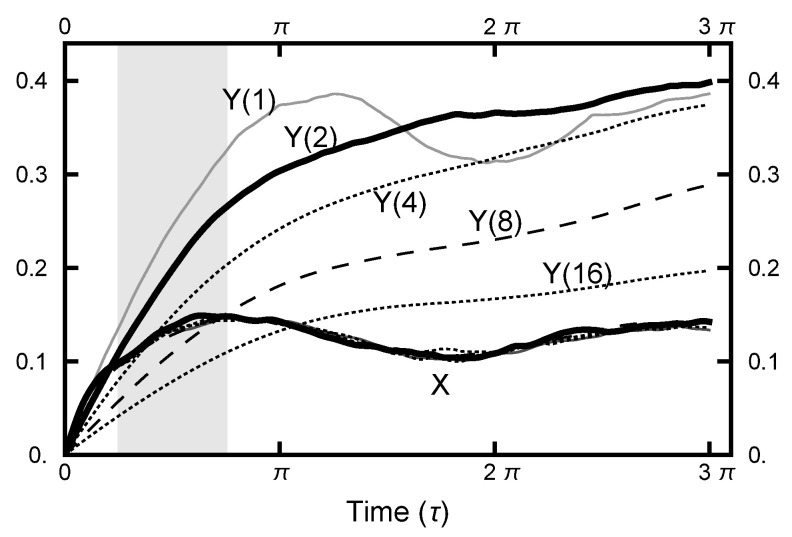
The correlation functions $d^{\left(x\right)}\left(\tau \right)$ and $A_{ou}^{\left(y\right)}$ for various runs. For all the runs, $A_{ou}^{\left(x\right)}=1$. From the top, the curves for $d^{\left(y\right)}\left(\tau \right)$ have the values of $A_{ou}^{\left(y\right)}$ of 1,2,4,8, and 16, respectively. The curves for $d^{\left(x\right)}\left(\tau \right)$ all lie on top of one other; only one is plotted.

To understand how the choice of $A_{ou}^{x}$ and $A_{ou}^{y}$ affect the sampling behavior, consider the plots of the correlation functions in Figure 7. As shown in the this figure and in Figure 5, the y-degree of freedom shows the floppiness that corresponds to the low energy cost of movement in that direction in either basin. The relative stiffness of motion in the x direction is a direct result of the restriction due to the narrow channel connecting the two basins. The floppiness (in the y direction) can be mathematically reduced by increasing the value of $A_{ou}^{y}$. For the chosen integration times (as designated as a gray rectangle in Figure 7), values $A_{ou}^{y}$ between four and eight put the correlation function $d^{\left(y\right)}\left(\tau \right)$ in the same range as $d^{\left(x\right)}\left(\tau \right)$. However, decreasing $A_{ou}^{x}$ below unity (not shown in the figures) does not alter the stiffness as its origin is due to the geometry of the connecting channel.

#### 3.2.4. MD Time Step Size (*h*)

**Figure 8:**
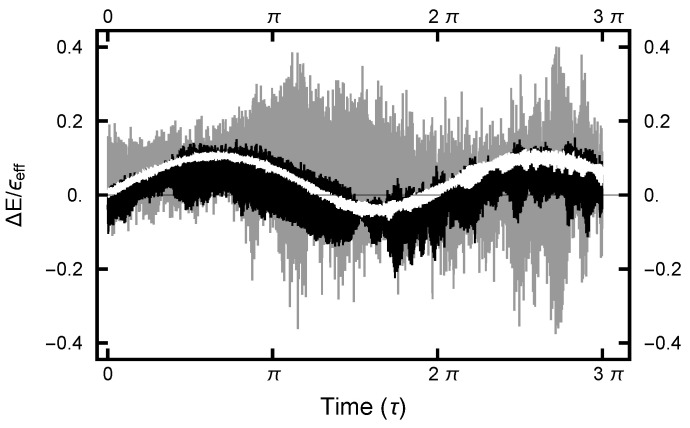
The effective-energy error plotted as a function of the MD integration time (τ). The three curves correspond to runs with values $A_{ou}^{\left(x\right)}=A_{ou}^{\left(y\right)}=1$. The gray curve gives the error for the run with h=0.002; the black curve, h=0.001; and the white curve, h=0.00067.

For the deterministic integration in the Molecular Dynamics (MD) step, a choice must be made for the size of the time increment *h*. The size of *h* must not be too large nor too small. In the first case, the errors become large and the acceptance rate becomes unacceptably small. In the second case, computational resources are wasted. Since the numerical algorithm is based on a Trotter–Strang [9,10] splitting, for small values of *h*, the energy error is bounded, as the energy of a “nearby” Hamiltonian (the shadow Hamiltonian) is conserved. As shown in Figure 8, for small *h*, the energy error oscillates as a function of integration time τ. In addition, because of the stochastic nature of the integrals, the curves are noisy, with the noise increasing as *h* increases.

**Figure 9:**
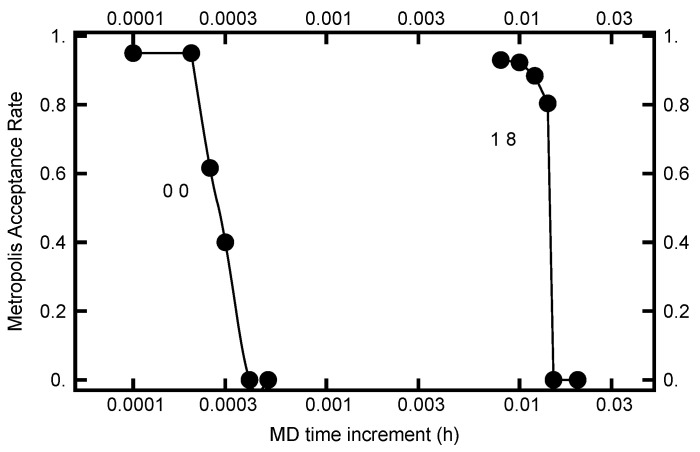
The Metropolis–Hastings acceptance rate for two runs. For the curve on the left, the parameters were $A_{OU}^{x}=A_{OU}^{y}=0$ and for the curve on the right, $A_{OU}^{x}=1$ and $A_{OU}^{y}=8$. For both runs, the number of molecular dynamics steps were chosen to be (1+η)π/(4h) with η being a uniformly distributed random number in the unit interval.

The value for *h* can be chosen by requiring the Metropolis–Hastings acceptance rate to be on the order of 80%. In Figure 9, this acceptance rate is plotted as a function of the time step *h*. As can be seen in the plot, the rate is a very steep function of *h*. Furthermore, when $A_{OU}^{x}=A_{OU}^{y}=0$, *h* must be less than 0.0002 to have a substantial acceptance rate, while when $A_{OU}^{x}=1$ and $A_{OU}^{y}=8$, *h* can be chosen almost two orders of magnitude larger. Evidently the acceptance rate is sensitive to *h* as the values of $A_{OU}^{x}$ and $A_{OU}^{y}$ change.

The above highlights the advantage of the novel algorithm presented here. By choosing the OU parameters, $A_{OU}^{x}$ and $A_{OU}^{y}$, to be unity, reasonably high acceptance rates can be achieved by using a value of *h* 10 times larger than when both OU parameters are zero. As shown in Figure 5, the nonlocality generated in the MD integration is only weakly dependent on the OU parameters, but does depend on the total MD integration time. Taken together, this then leads to a corresponding factor of 10 decrease in the computational effort by choosing the OU parameters to be unity. As shown in Figure 9, larger values of *h* can be used when larger OU parameters are used, leading to even fewer MD steps being necessary to attain the same MD integration time. This translates into a speedup of a factor of 10 or more, showing the power of picking nonzero values for the OU parameters and using the corresponding “optimal” value for *h*.

## 4. Path Sampling

**Figure 10:**
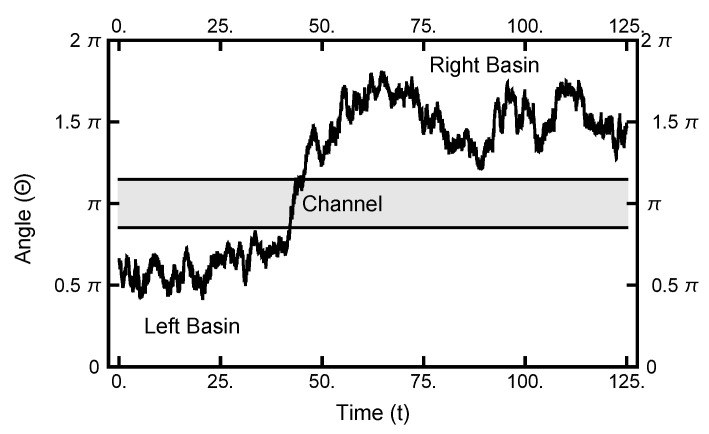
The one-dimensional representation of a typical path. The angle Θ plotted as a function of time. See Figure 2 for the definition of Θ. At t=0, the particle starts out in the left basin, makes its way through the narrow channel at t≈50, and ends in the right basin.

As seen in Figure 2 and Figure 3, in equilibrium, a particle will spend a small fraction of time in the narrow channel. Thus, the expectation is that a transition path would consist of motion in one basin or the other with a short transition while in the narrow channel. In Figure 10, a typical path is displayed: it starts in the left basin, remaining in it for under 25% of the path length, making a transition, remaining in the right basin for approximately the last 75% of the path. The boundary conditions influence the shape of the path, although the time spent in the narrow channel can change during the sampling procedure. The shown path is the end result of a simulation using $A_{OU}^{x}=1$ and $A_{OU}^{y}=8$ after 100,000 Metropolis–Hastings steps.

**Figure 11:**
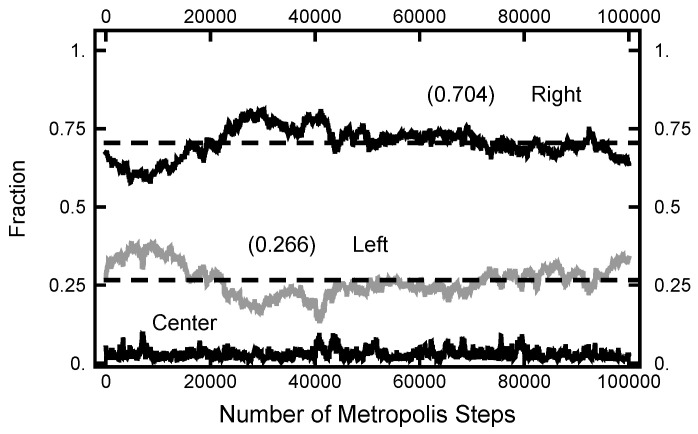
Results for a calculation with $A_{OU}^{x}=1$ and $A_{OU}^{y}=8$. The bottom (black) curve is the fraction of the path with |x|<1/2; the middle (gray) curve is the fraction of the path with x<−1/2; and the top (black) curve is the fraction of the path with x>1/2.

**Figure 12:**
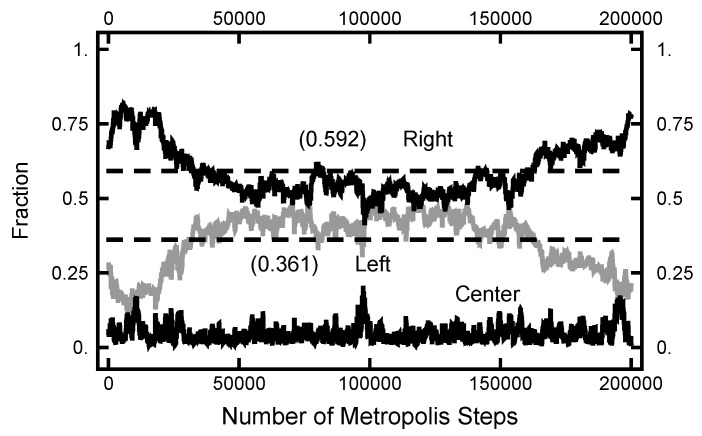
Results for a calculation with $A_{OU}^{x}=1$ and $A_{OU}^{y}=4$. The bottom (black) curve is the fraction of the path with |x|<1/2; the middle (gray) curve is the fraction of the path with x<−1/2; and the top (black) curve is the fraction of the path with x>1/2.

In Figure 11, a summary of the evolution of this simulation is plotted. For these values of $A_{OU}$, on average, the particle spends about 70% of the time in the right basin. The variation of this percentage changes very little (only a few percent) over the course of the simulation run. In Figure 12, a similar plot is shown for the parameters $A_{OU}^{x}=1$ and $A_{OU}^{y}=4$ and 200,000 Metropolis–Hastings steps, twice as long as used for the results shown in Figure 11. The pictures are similar, except that the mean percentages are slightly shifted, and the variation about the mean is larger in the second case. The smaller variation in the first case is due to the larger value of $A_{OU}^{y}$, which dampens the oscillations, the floppiness, in the *y* component. With either parameter set, the path sampling calculations correctly infer that the free energy of the right basin is larger that of the left basin.

**Figure 13:**
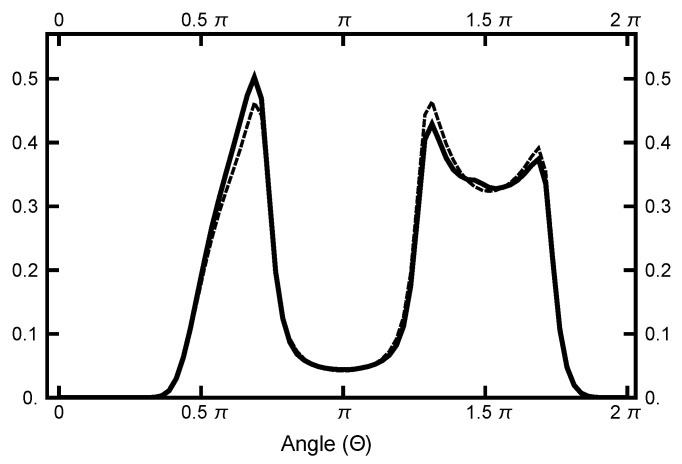
Results for a calculation with $A_{OU}^{x}=1$ and $A_{OU}^{y}=4$. The solid black curve is the histogram of the $\bar{P}\left(\Theta \right)$ for the 200,000 Metropolis steps pictured above in Figure 12. The dashed line represents the histogram that corresponds to the equilibrium distribution.

Now, consider Figure 13, where the information for the second simulation run is summarized. Here, the normalized histogram is plotted for the run with $A_{OU}^{x}=1$ and $A_{OU}^{y}=4$. Note that the comparison with the equilibrium histogram is quite close. This closeness indicates that the calculated ensemble of paths accurately probes the potential shape of each basin.

**Figure 14:**
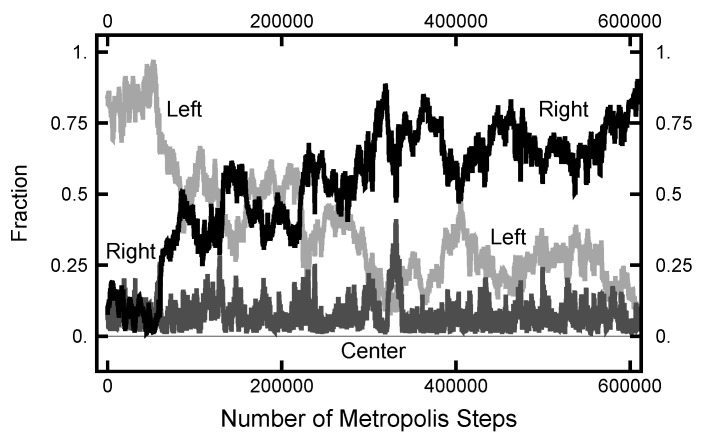
Results for a calculation with $A_{OU}^{x}=A_{OU}^{y}=1$. The black curve is the fraction of the path with x>1/2; the light gray curve is the fraction of the path with x<−1/2; and the bottom (dark gray) curve is the fraction of the path with |x|<1/2.

The results shown in Figure 11 and Figure 12 used an initial path where the fraction spent in the left basin was less than the fraction spent in the right. This was chosen because the relative fractions were approximately what was expected from their equilibrium values. For the next calculation, the starting path had a value for the fraction spent in the left well of ≈80%, much larger than its equilibrium value. In Figure 14, the evolution of the right and left fractions is shown as a function of the algorithmic steps. In this simulation, $A_{OU}^{x}=A_{OU}^{y}=1$ and the MD time step was h=0.003. As indicated, it took about $10^{5}$ steps before the left and right fractions became roughly equal and twice that many steps before the right fraction became significantly larger that the left. The change in the fraction was due to the movement of the time at which the transition took place and not the creation of a new pair of transitions. One does see an incipient attempt at the creation of a new pair of transitions at the ≈325,000th step of the simulation. This attempt is signaled by the spike in the fraction of the path that is spent in the narrow channel, shown as the dark grey curve.

## 5. Continuous-Time Limit

**Figure 15:**
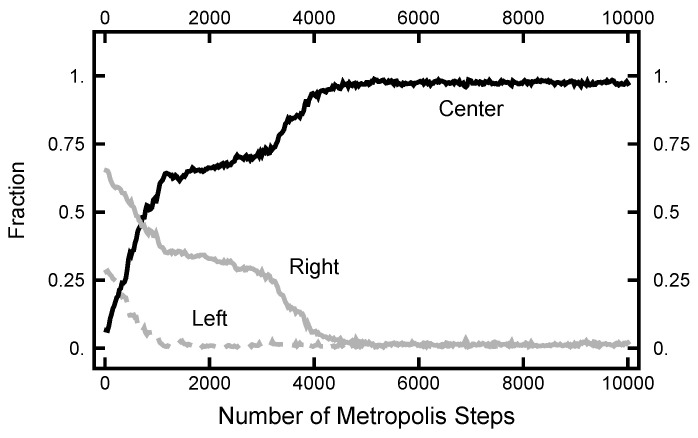
Results for a calculation with $A_{OU}^{x}=1$ and $A_{OU}^{y}=4$ using Equation (A11) as the effective Hamiltonian. The black curve labeled “Center” is the fraction of the path with |x|<1/2; the solid gray curve is the fraction of the path with x>1/2; and the dashed gray curve is the fraction of the path with x<−1/2.

**Figure 16:**
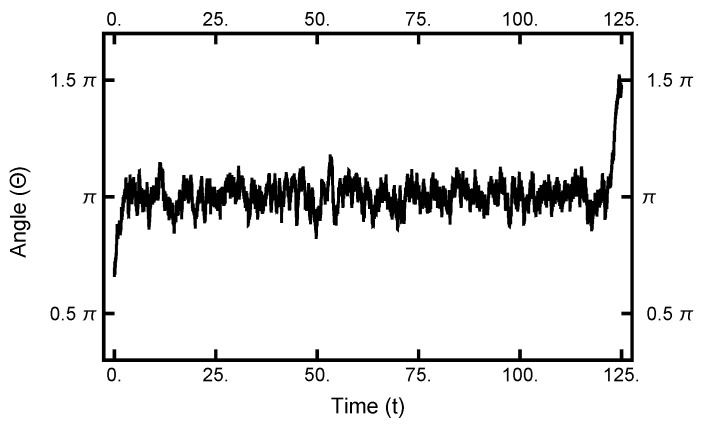
Results for a calculation with $A_{OU}^{x}=1$ and $A_{OU}^{y}=4$ using Equation (A11) as the effective Hamiltonian. This is qualitatively different from the path plotted in Figure 10.

Now, I turn to the calculations using the continuous-time limit of the OM functional [13,14]. The Radon–Nikodym derivative is manipulated using the Girsanov theorem and Ito’s lemma. Displayed in Equation (A11), this is denoted as the Ito–Girsanov measure. As shown in Figure 15, the results of a simulation using the Ito–Girsanov measure gave unphysical results. As shown in Figure 16, the paths converge very quickly to objects that look similar to noisy versions of the Most Probable Path (MPP). Notice that this behavior happens after only 5000 hybrid steps. The MPP reflects the maximum of the measure and is dominated by the maxima of the Laplacian (of the physical potential). In the narrow channel, the Laplacian reflects the large curvature due to the confining sides. For calculations using the discrete form of the OM functional, Equation (A4), a very different behavior is found, as shown in Figure 11. This unphysical behavior is similar to what was found before [7,8], where the cause was traced back to a buildup of correlations that were inconsistent with the assumption in the original SDE that the noise is white and uncorrelated with the particle position.

## 6. Discussion

This work is related to the recent work of Korol et al. [15], who explored using a similar approach and applied it to path-integral molecular dynamics. In that work, the authors suggested that one should use mollified forces. This force mollification is a frequency-dependent factor that limits the effects of high-frequency internal modes and yet keeps intact the low-frequency motion. In this current work, this task is performed by the mass operator, $M\;=\;L+A_{ou}^{2}𝟙$. Here, $A_{ou}$ can be adjusted to change the crossover between low- and high-frequency behavior. At high frequencies, the modes revert to the free-particle motion. This reversion is due to the effects of the *L* in the mass operator, which dominates at high frequencies. In the novel method described here, this “force mollification” is folded into a unified framework, incorporating its effects in a self-consistent manner.

If, instead of the dynamics, one is interested in highlighting particular modes, one can choose $A_{ou}$ differently for each mode. In particular, when working in normal mode coordinates, one can choose $A_{ou}$ individually to minimize the size of Δv in Equation (13), thereby reducing the numerical errors in the integration.

## 7. Conclusions

A novel HMC procedure for sampling paths was presented here. This new procedure is much more efficient than previous methods, resulting in an order of magnitude speedup for the two-dimensional problem explored. The decrease in computational effort comes from increasing the size of the MD time increment *h* by at least an order of magnitude. The novelty in the procedure lies in the choice of the mass matrix and concomitantly the velocities that form an OU bridge. Using this new procedure, I successfully examined transitions across an entropic barrier and found that the method can determine the relative free energy of the two basins that are not separated by an energy barrier.

Again, using the Ito–Girsanov measure was found to give unphysical results. The sampling procedure, for the two-dimensional problem examined, converged to paths that look like noisy versions of the MPP [16] (most probable path), where the vast majority of the path was trapped in the narrow channel that connected the probability basins. Clearly, the source for this unphysical feature is the Laplacian (of the physical potential) in the formula for the measure.

This new algorithm is also useful for calculations using path-integral molecular dynamics [15] where the effective Hamiltonian has a similar form to that studied here. The novel procedure presented here naturally folds the “mollification” of forces into the HMC formalism by a modification of the mass matrix. Thus, this novel procedure includes this mollifying effect in a self-consistent manner. The parameters $\left\{A_{ou}\right\}$ in this novel method are able to be tuned individually for each degree of freedom to emphasize different physical effects.

In addition, this method is able to be adapted for use with the OBABO integration scheme [17] or with the second-order Langevin method [18]. 

## Data Availability

Not applicable.

## Appendix A. Onsager–Machlup Functionals

In this Appendix, the algebraic forms of the OM functionals are delineated. For simplicity, the one-dimensional forms of the equations are written below; the multi-dimensional generalization is straight forward. First, recognize that different discretizations of the SDE (Equation (1)) result in different forms of the OM function [19]. Here, two are considered: the Euler–Maruyama and midpoint methods. The former is given by:

(A1) $$x_{n+1}=x_{n}+F\left(x_{n}\right)\;\Delta t\;+\;\sqrt{2\;\epsilon \;\Delta t\;}\;\xi_{n+1}$$

where $\xi_{n}$ is a random Gaussian variate with mean zero and unit variance and Δt is the time increment. The original Onsager–Machlup functional is based on this expression. If one iterates Equation (A1) $N_{t}$ times, one obtains a sequence of points $\left|x>=\{x_{0},\;x_{1},\;x_{2},\;x_{3},\;x_{4},\;\cdots \;x_{n_{t}}\right\}$, with a probability:

(A2) $$P_{EM}\;=\;{\left(2\;\pi \right)}^{-n_{t}/2}\;\prod_{n=1}^{n_{t}}\exp\;\left(-\frac{1}{2}\xi_{n}^{2}\right)$$

Onsager and Machlup recognized that one can rewrite this probability distribution in terms of the path positions as $P_{EM}\;\propto \exp\;(-I_{OM})$ with:

(A3) $$I_{OM}=\frac{\Delta t}{2\epsilon }\sum_{n=1}^{n_{t}}\;\frac{1}{2}{\left(\frac{x_{n+1}-x_{n}}{\Delta t}-F\left(x_{n}\right)\right)}^{2}$$

Defining $\epsilon_{eff}=2\;\epsilon /\Delta t$ as an effective temperature:

$$P_{EM}\;\propto \exp\;\left(\;\;-\;\frac{H_{EM}}{\epsilon_{eff}}\right)$$

with:

$$H_{EM}=\sum_{n=1}^{n_{t}}\;\frac{1}{2}{\left(\frac{x_{n+1}-x_{n}}{\Delta t}\right)}^{2}-\left(\frac{x_{n+1}-x_{n}}{\Delta t}\right)\;F\left(x_{n}\right)+\frac{1}{2}F{\left(x_{n}\right)}^{2}$$

which can be rewritten as:

(A4) $$\begin{array}{c} H_{EM}=\frac{1}{2\;\Delta t^{2}}\sum_{n=2}^{n_{t}-1}x_{n}\left(2\;x_{n}-x_{n+1}-x_{n-1}\right)+\sum_{n=1}^{n_{t}}\left(\frac{1}{2}F{\left(x_{n}\right)}^{2}-\left(\frac{x_{n+1}-x_{n}}{\Delta t}\right)\;F\left(x_{n}\right)\right) \end{array}$$

Here, it is convenient to use the bra-ket mathematical notation. Specifically:

$$\sum_{n}\sum_{m}\;x_{n}\;B_{n,\;m}\;x_{m}=\langle x\;|\;B\;|\;x\rangle .$$

By rearranging the order in the sum, the first term in Equation (A4) can be transformed to:

(A5) $$\begin{array}{c} \frac{1}{2}\;\sum_{n=1}^{n_{t}}{\left(\frac{x_{n+1}-x_{n}}{\Delta t}\right)}^{2}=\frac{1}{2\;\Delta t^{2}}\sum_{n=2}^{n_{t}-1}x_{n}\left(2\;x_{n}-x_{n+1}-x_{n-1}\right)=\frac{1}{2}\langle x\;|\;L\;|\;x\rangle , \end{array}$$

where *L* is a symmetric, tridiagonal, positive definite matrix. The last term in Equation (A4) is denoted as $\Phi_{EM}\left(\left\{x\right\}\right)$. The effective Hamiltonian can be written as:

(A6) $$H_{EM}\;=\;\frac{1}{2}\langle x\;|\;L\;|\;x\rangle +\Phi_{EM}\left(\left\{x\right\}\right)$$

Another discretization of the SDE (Equation (1)) uses the Stratonovich midpoint [20] integration and is written as:

(A7) $$x_{n+1}=x_{n}+F\left(x_{mp}\right)\;\Delta t\;+\;\sqrt{2\;\epsilon \;\Delta t\;}\;\xi_{n+1}$$

with $x_{mp}=\left(x_{n+1}+x_{n}\right)/2$. The effective Hamiltonian is a bit more complicated because of the Jacobian and again can be put in the form:

(A8) $$H_{mp}\;=\;\frac{1}{2}\langle x\;|\;L\;|\;x\rangle +\Phi_{mp}\left(\left\{x\right\}\right).$$

The second term in the equation above is:

(A9) $$\begin{array}{c} \Phi_{mp}\left(\left\{x\right\}\right)=-\frac{2\;\epsilon }{\Delta t}\;\sum_{n=1}^{n_{t}}\;\ln\left(\det J_{n}\right)\;+\;\sum_{n=1}^{n_{t}}\left(\frac{1}{2}F{\left(x_{mp}\right)}^{2}-\left(\frac{x_{n+1}-x_{n}}{\Delta t}\right)\;F\left(x_{mp}\right)\right) \end{array}$$

where $J_{n}$ is the Jacobian matrix, whose elements are:

(A10) $$J_{\left(\alpha ,\beta \right)}\;\propto \;\delta_{\alpha ,\beta }+\frac{\Delta t}{2}\frac{\;\partial^{2}U}{\partial x_{\alpha }\;\partial x_{\beta }}$$

and where the Jacobian $J_{n}$ is evaluated at the midpoint.

Lastly, the continuous-time limit of Equations (A6) and (A8) can be written as:

(A11) $$H_{IG}\;=\;\frac{1}{2}\langle x\;|\;L\;|\;x\rangle +\Phi_{IG}\left(\left\{x\right\}\right)$$

with:

$$\Phi_{IG}\left(\left\{x\right\}\right)=\sum_{n=1}^{n_{t}}\;G\left(x_{n}\right)$$

where:

(A12) $$G\left(x_{n}\right)=\frac{1}{2}\;F^{2}\left(x_{n}\right)\;-\;\epsilon \;\;U^{\prime \prime }\left(x_{n}\right)$$

in one dimension. In higher dimensions, the Laplacian of *U* replaces $U^{\prime \prime }\left(x_{n}\right)$.

## Appendix B. Constructing Ornstein–Uhlenbeck Bridges

The following describes the method used to construct Ornstein–Uhlenbeck (OU) bridges. An OU bridge is an OU process that begins and ends at the origin. Consider the standard OU process for a variable $Z_{i}$ given by the following SDE:

(A13) $$dZ_{t}=-A\;Z_{t}\;dt+\sqrt{2\;\epsilon \;}\;dW_{t}$$

with *A* being the OU parameter and ϵ being the temperature.

To form a realization of an OU process, we use the close relationship between HMC and the Metropolis Adjusted Langevin Algorithm (MALA). To sample the Boltzmann distribution for a harmonic potential, the Hamiltonian is augmented by auxiliary variables, which can be viewed as momenta, conjugate to the position variables. In one dimension, this is given by:

(A14) $$H=\frac{1}{2}p^{2}+\frac{1}{2}A\;x^{2}$$

The equations of motion are then:

(A15) $$\frac{dx}{dt}\;=\;p\;\mathrm{and}\;\frac{dp}{dt}\;=\;-A\;x$$

The HMC procedure is first to form the momentum $p_{0}=\sqrt{\epsilon }\;\xi$ where ϵ is the temperature and ξ is a Gaussian random variate with mean zero and unit variance.

If one integrates the equations of motion over a time *h* and uses the velocity-Verlet approximation, one obtains:

(A16) $$x_{1}=x_{0}+p_{0}\;h-\frac{A}{2}x_{0}\;h^{2}$$

which becomes:

(A17) $$x_{1}-x_{0}=-A\;x_{0}\;\Delta t+\sqrt{2\;\epsilon \;\Delta t}\;\xi$$

with $\Delta t=h^{2}/2$. This is then the Euler–Maruyama [21] formula for the OU process. Within the one-step HMC, one uses the Metropolis–Hastings step to accept or reject the proposed move:

(A18) $$\left\{x_{0},\;p_{0}\right\}\to \left\{x_{1},\;p_{1}=p_{0}\;-\;\sqrt{\frac{\Delta t}{2}\;}\;A\;\left(x_{0}+x_{1}\right)\right\}.$$

Such a move may be rejected if the energy error is too large.

However, the equations of motion above can be integrated exactly, which gives:

(A19) $$x_{1}=x_{0}\;\cos\sqrt{2\;A\;\Delta t}\;+\;\xi_{0}\;\sqrt{\frac{\epsilon }{A\;}}\;\sin\sqrt{2\;A\;\Delta t}.$$

In such a method, no proposed moves will be rejected in the Metropolis–Hastings step. Thus, these steps can be combined to obtain

(A20) $$x_{i+1}=x_{i}\;\cos\sqrt{2\;A\;\Delta t}\;+\;\xi_{i}\;\sqrt{\frac{\epsilon }{A\;}}\;\sin\sqrt{2\;A\;\Delta t}.$$

To first order in Δt, the above agrees with other expressions for forming a realization of an OU process. The advantage of the above form is that for any size of Δt, the path asymptotically approaches the target Boltzmann (Gaussian) distribution.

Now, the ending point of the OU bridge needs to be addressed. The ending point of an OU bridge is also zero, but this is not the case for a typical realization of an OU process. To form a bridge, two OU processes can be combined by the following procedure. Assume that $Z^{\left(1\right)}$ and $Z^{\left(2\right)}$ are two different finite realizations of Equation (A13); both starting at the origin with endpoints $z_{N}^{\left(1\right)}$ and $z_{N}^{\left(2\right)}$, respectively. The linear combinations:

(A21) $$Z_{\pm }^{\left(3\right)}=\pm \left(Z^{\left(1\right)}\;\cos\kappa +Z^{\left(2\right)}\;\sin\kappa \right)$$

are also realizations of the same OU process. Then, the angle κ can be chosen by $\tan\kappa =-z_{N}^{\left(1\right)}/z_{N}^{\left(2\right)}$, which makes the endpoints of $Z_{\pm }^{\left(3\right)}$ be $z_{N}^{\left(3\right)}=0$. With this choice of κ, the OU processes $Z_{\pm }^{\left(3\right)}$ are OU bridges. Another way exists in the literature [22] for forming OU bridges. However, I found it to be numerically unstable due to the need to calculate the ratios of hyperbolic sines with large arguments.

## Appendix C. BAB Splitting: Numerical Integration

In this section, the numerical algorithm is written down for the case when the BAB splitting is used. The method is schematically described in Equation (A22).

(A22) $$\;\;\;\;\left(\begin{array}{c} q_{0}\\ v_{0} \end{array}\right)\overset{B(h/2)}{\underset{\;\mathrm{Half}\;\mathrm{step}}{\Rightarrow }}\left(\begin{array}{c} q_{0}\\ v_{H} \end{array}\right)\overset{A(h)\;}{\underset{\;\mathrm{Full}\;\mathrm{step}}{\Rightarrow }}\left(\begin{array}{c} q_{1}\\ w_{H} \end{array}\right)\overset{B(h/2)}{\underset{\;\mathrm{Half}\;\mathrm{step}}{\Rightarrow }}\left(\begin{array}{c} q_{1}\\ v_{1} \end{array}\right)$$

Half step, B: $\left\{q_{0},\;v_{0}\right\}\Rightarrow \left\{q_{0},\;v_{H}\right\}$ with $M=(L+A_{ou}^{2}𝟙)$

(A23) $$M\;\frac{d}{dt}|v>=-\left(\;|\phi \left(q\right)>-A_{ou}^{2}|q>\right)\;\Rightarrow M\;|\Delta v_{0}>=-\frac{h}{2}\;|Big(\;|\phi \left(q_{0}\right)>-A_{ou}^{2}|q_{0}>)$$

with $|\Delta v_{0}>=\left|v_{H}>-\;\right|v_{1}>$. Full step, A: $\left\{q_{0},\;v_{H}\right\}\Rightarrow \left\{q_{1},\;w_{H}\right\}$ with θ=h

(A24) $$\frac{d}{dt}\left|q>=\;\;|v>\;\Rightarrow \;\right|q_{1}>=\;\;\cos\;(\theta )\;\left|q_{0}>+\sin\;(\theta )\;\right|v_{H}>$$

(A25) $$\frac{d}{dt}\left|v>=-|q>\;\Rightarrow \;\right|w_{H}>=-\sin\;(\theta )\;\left|q_{0}>+\cos\;(\theta )\;\right|v_{H}>$$

Half step, B: $\left\{q_{1},\;w_{H}\right\}\Rightarrow \left\{q_{1},\;v_{1}\right\}$ with $M=(L+A_{ou}^{2}𝟙)$

(A26) $$M\;\frac{d}{dt}\left|v>=-\left(\;|\phi \left(q\right)>-A_{ou}^{2}|q>\right)\;\Rightarrow M\;\right|\Delta v_{1}>=-\frac{h}{2}\;\left(\;|\phi \left(q_{1}\right)>-A_{ou}^{2}|q_{1}>\right)$$

with $|\Delta v_{1}>=\left|v_{1}>-\right|w_{H}>$.

## Appendix D. BAB Splitting: Energy Error

In the BAB splitting, the error in the effective energy is:

(A27) $$\begin{array}{c} \begin{array}{cc} \Delta E^{\left(01\right)}= & \;\Phi \left(q_{1}\right)-\Phi \left(q_{0}\right)-\frac{1}{2}\langle q_{1}\;\left|\;A_{ou}^{2}\;\right|\;q_{1}\rangle +\frac{1}{2}\langle q_{1}\;\left|\;A_{ou}^{2}\;\right|\;q_{0}\rangle \\ + & \;\frac{1}{2}\langle v_{1}\;\left|\;M\;\right|\;v1\rangle -\frac{1}{2}\langle v_{0}\;\left|\;M\;\right|\;v_{0}\rangle +\frac{1}{2}\langle q_{1}\;\left|\;M\;\right|\;q1\rangle -\frac{1}{2}\langle q_{0}\;\left|\;M\;\right|\;q_{0}\rangle \end{array} \end{array}$$

which simplifies to:

(A28) $$\begin{array}{c} \begin{array}{cc} \Delta E^{\left(01\right)}= & \;\Phi \left(q_{1}\right)-\Phi \left(q_{0}\right)-\frac{1}{2}\langle q_{1}+q_{0}\;\left|\;A_{ou}^{2}\;\right|\;q_{1}-q_{0}\rangle \\ + & \;\frac{1}{2}\langle v_{1}+w_{H}\;\left|\;M\;\right|\;v_{1}-w_{h}\rangle +\frac{1}{2}\langle v_{H}+v_{0}\;\left|\;M\;\right|\;v_{H}-v_{0}\rangle . \end{array} \end{array}$$

One can express this in terms of $q_{0}$ and $q_{1}$, using Equations (A23) and (A26) and:

(A29) $$\begin{array}{ccc} & & |v_{H}>=-\left|q_{0}>\;\cot\theta +\right|q_{1}>\csc\theta \end{array}$$

(A30) $$\begin{array}{ccc} & & |w_{H}>=\;\;\left|q_{1}>\;\cot\theta -\right|q_{0}>\csc\theta \end{array}$$

In the work by Beskos et al. [3], a similar method was constructed for the case where $A_{ou}=0$. In the middle step of the numerical integration (Full Step A), the Crank–Nicolson method was used. This changes the rotation angle in Equations (A24) and (A25) to $\theta =\sin^{-1}\;\left(4\;h/(4+h^{2})\right)$. This in turn modifies the solutions for $v_{H}$ and $w_{H}$ used in Beskos et al., namely:

(A31) $$\begin{array}{ccc} & & \;|v_{H}>\;=\frac{1}{h}\;\left(|q_{1}>-|q_{0}>\right)+\frac{h}{4}\;\left(|q_{1}>+|q_{0}>\right)\; \end{array}$$

(A32) $$\begin{array}{ccc} & & \;|w_{H}>=\frac{1}{h}\;\left(|q_{1}>-|q_{0}>\right)-\frac{h}{4}\;\left(|q_{1}>+|q_{0}>\right)\; \end{array}$$
